# A proteoglycan-based topical treatment for hair greying: *in vitro* antioxidant and pro-melanogenic effects

**DOI:** 10.3389/fmed.2025.1734454

**Published:** 2026-01-14

**Authors:** David Vega Diez, Eva María Arias, Nuria Floriach, Estela Bravo

**Affiliations:** 1 Dermatology Department, Servicio De Dermatología, Universidad de Alcalá, Hospital Universitario Principe de Asturias, Alcala de Henares, Spain; 2 R&D Department, Laboratorio Genové, Barcelona, Spain

**Keywords:** canities, hair aging, hair greying, senescent alopecia, trichology

## Abstract

**Introduction:**

Hair greying (canities) is a common and impactful feature of hair aging, primarily driven by oxidative stress, mitochondrial dysfunction, and progressive impairment of melanocyte function. Reactive oxygen species disrupt key melanogenic pathways, including Wnt/β-catenin signaling and microphthalmia-associated transcription factor (MITF) activity. Given the central role of oxidative and inflammatory processes in pigment loss, antioxidant and melanocyte-stimulating strategies represent promising approaches to delay hair greying.

**Methods:**

The present study evaluated the *in vitro* antioxidant, anti-inflammatory, and pro-melanogenic effects of PP-PTKL (Pilopeptan^®^ Woman Proteokel), a proteoglycan-based topical formulation. Total antioxidant capacity was assessed using a copper reduction assay. Pro-melanogenic activity was evaluated in Normal Human Epidermal Melanocytes (NHEM) under Wnt/β-catenin inhibition induced by FH535. Anti-inflammatory effects were assessed through histamine release in mast cells and by measuring IL-1Ra and S100A8/9 levels using ELISA.

**Results:**

PP-PTKL significantly restored MITF expression in a dose-dependent manner in human melanocytes subjected to Wnt/β-catenin pathway inhibition, with increases of 43.1% at 1% and a 3.7-fold increase at 5%. The formulation exhibited strong synergistic antioxidant activity, with total antioxidant capacity nearly four times higher than the sum of its individual components. In addition, histamine release was significantly reduced in mast cells, while IL-1Ra and S100A8/9 levels were significantly decreased compared with placebo, indicating attenuation of inflammation-associated oxidative stress.

**Conclusion:**

PP-PTKL demonstrates combined pro-melanogenic, antioxidant, and anti-inflammatory activity *in vitro*, supporting its potential as a preventive or therapeutic strategy for hair greying. Further *in vivo* and clinical studies are warranted to confirm these findings.

## Introduction

Hair aging has gained increasing relevance with rising global life expectancy and greater cosmetic awareness. This physiological process is characterized by reduced hair growth, decreased hair shaft diameter, alterations in cuticle structure, impaired lipid production and changes in pigmentation that result in hair greying, also known as canities (1). Hair aging is a multifactorial process involving genetic predisposition, scalp conditions, as well as external factors such as cosmetic practices, environmental pollutants, and ultraviolet (UV) radiation exposure (2, 3). This changes affect self-image and quality of life (4). Canities, resulting from the progressive decline in melanin synthesis within melanocyte melanosomes (5, 6), also plays a major role in social perception, as both men and women with a higher prevalence of grey hair are commonly perceived as older (4).

The free radical theory of aging remains the most accepted theory for hair greying: reactive oxygen species (ROS) damage DNA, proteins and lipids, progressively impairing melanocyte and melanocyte stem cell (MSC) function (7). Under normal physiological conditions a robust cellular antioxidant mechanisms prevent ROS-induced damage, but these defenses weaken with age, leading to oxidative damage (6, 8). This impaired maintenance of melanocytic cells alter proper activation of the Wnt/β-catenin signaling (9–11). Wnt signaling regulates hair pigmentation by controlling the transcription of the tyrosinase (TYR) gene, which encodes the rate-limiting enzyme in melanin synthesis (12). This regulation occurs both directly and through Microphthalmia-associated Transcription Factor (MITF) (12–14), the master regulator of melanogenesis. MITF is essential for melanocyte differentiation and survival, and its dysregulation, as well as the downregulation of Wnt/β-catenin leads to hypopigmentation (8, 15–18). MITF activity is also modulated by oxidative stress and by members of the S100 protein family, which play critical roles in proliferation, inflammation, and apoptosis (19–21).

Given this connection between oxidative stress and melanocyte dysfunction, antioxidant-based interventions and modulation of inflammatory pathways may represent promising approaches to delay hair greying (22). Considering the regulatory role of proteoglycans in follicular homeostasis (23) the present study investigates the *in vitro* antioxidant activity and melanogenesis-stimulating potential of PP-PTKL (*Pilopeptan^®^ woman proteokel*), a novel topical formulation containing biomimetic peptides of proteoglycans (decorin and versican), caffeine, *pyrus malus* extract, cystine, methionine, Ginseng extract and inositol, with the aim of advancing therapeutic strategies for age-related changes in hair pigmentation. In addition, the ability of *Pyrus malus* extract to reduce inflammatory markers associated with ROS generation—such as histamine, interleukin-1 receptor antagonist (IL-1Ra), and S100A8/9—was also assessed.

## Material and methods

### Total antioxidant capacity (TAC) assay

The total antioxidant capacity (TAC) of the tested ingredients was assessed using a commercially available TAC assay kit (Sigma-Aldrich, USA), based on the ability of antioxidant compounds to reduce Cu^2+^ to Cu^+^. The reduced Cu^+^ forms a colored complex with a chromogenic reagent, with color intensity directly proportional to the antioxidant capacity of the sample.

Absorbance was measured at 570 nm using a SpectraMax ABS Plus microplate reader (Molecular Devices, USA). after incubation at room temperature for 10 min. The assay was performed in 96-well plates following the manufacturer’s instructions. A Trolox standard curve (1.5–1,000 μM) was generated using serial dilutions of a 1 mM Trolox solution, with the zero-Trolox condition used as the blank control. Antioxidant capacity was expressed as Trolox equivalents (μM) derived from the standard curve.

The antioxidant activity of individual formulation components (including proteoglycans, caffeine, cystine/methionine/inositol, and *Pyrus malus* fruit extract) was evaluated and compared with the complete formulation (PP-PTKL). All samples and standards were analyzed in triplicate.

### *In vitro* assay of functional effects on hair graying in human melanocytes

The pro-melanogenic activity of the test product was evaluated in Normal Human Epidermal Melanocytes (NHEM) Cells were cultured under standard conditions and exposed for 24 h to the test formulation at sub-cytotoxic concentrations. To mimic pigmentation impairment associated with hair greying, melanocytes were co-treated with FH535 (100 μM), a selective inhibitor of Wnt/β-catenin signaling known to downregulate microphthalmia-associated transcription factor (MITF).

At the end of the incubation period, total RNA was isolated, reverse-transcribed into complementary DNA (cDNA), and relative MITF gene expression was quantified by real-time quantitative PCR (RT-qPCR) using a QuantStudio™ 5 Real-Time PCR System (Applied Biosystems). MITF expression levels were normalized to housekeeping genes and expressed relative to control conditions.

All experiments were performed in triplicate. Statistical analysis was carried out using one-way analysis of variance (ANOVA) with Dunnett’s multiple comparison test, and statistical significance was defined as *p* < 0.05.

### Histamine, IL1-Ra and S100A8/9

The effect of the active ingredient on histamine release was evaluated *in vitro* using murine mast cells (MC/9). Cells were cultured under standard conditions in Dulbecco’s Modified Eagle Medium (DMEM). After 24 h of incubation, cells were pre-treated with the test ingredient for 2 h, followed by stimulation with the calcium ionophore A23187, a well-known inducer of histamine release.

After incubation, culture supernatants were collected and histamine levels were quantified using a commercial Histamine Enzyme Immunoassay (EIA) kit, according to the manufacturer’s instructions.

The effect on inflammatory biomarkers was additionally assessed ex vivo in human volunteers. Skin surface samples were collected using swabs from the forehead at the scalp edge. Levels of interleukin-1 receptor antagonist (IL-1Ra) and S100A8/9 were quantified using commercially available ELISA kits following the manufacturers’ protocols. Absorbance was measured using a SpectraMax 340PC microplate reader (Molecular Devices). All measurements were performed under standardized conditions.

## Results

### Effect on MITF expression

**Figure 1:**
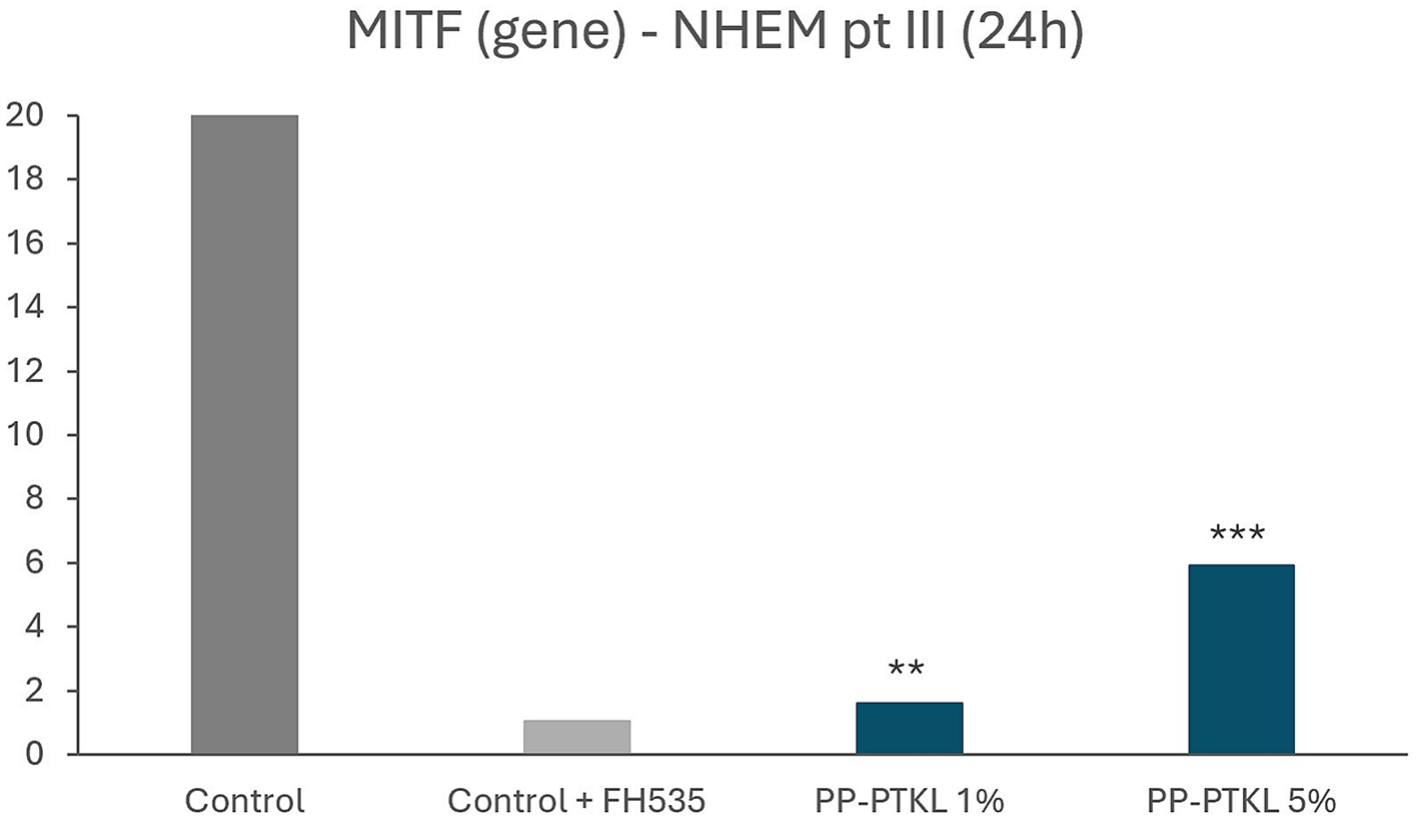
Effect of the tested samples on pigmentation-related genes expression. Bar graph representing MITF expression levels, after 24 h of simultaneous exposure to 100 μM FH535 and the test sample at the doses indicated in normal human epidermal melanocytes. Asterisks (*) over bars indicate statistical significance vs. Control.

Regarding the melanogenesis assay, FH535 inhibition reduced MITF expression by 97.3%, confirming effective Wnt pathway inhibition and the establishment of an anti-pigment model. Co-treatment with PP-PTKL significantly counteracted FH535-induced MITF suppression in a dose-dependent manner. At 1% concentration, PP-PTKL increased MITF expression by 43.1%, while a more pronounced effect was observed at 5%, with a 3.7-fold (370%) increase compared to FH535-treated controls (Figure 1).

### Antioxidant capacity

**Figure 2:**
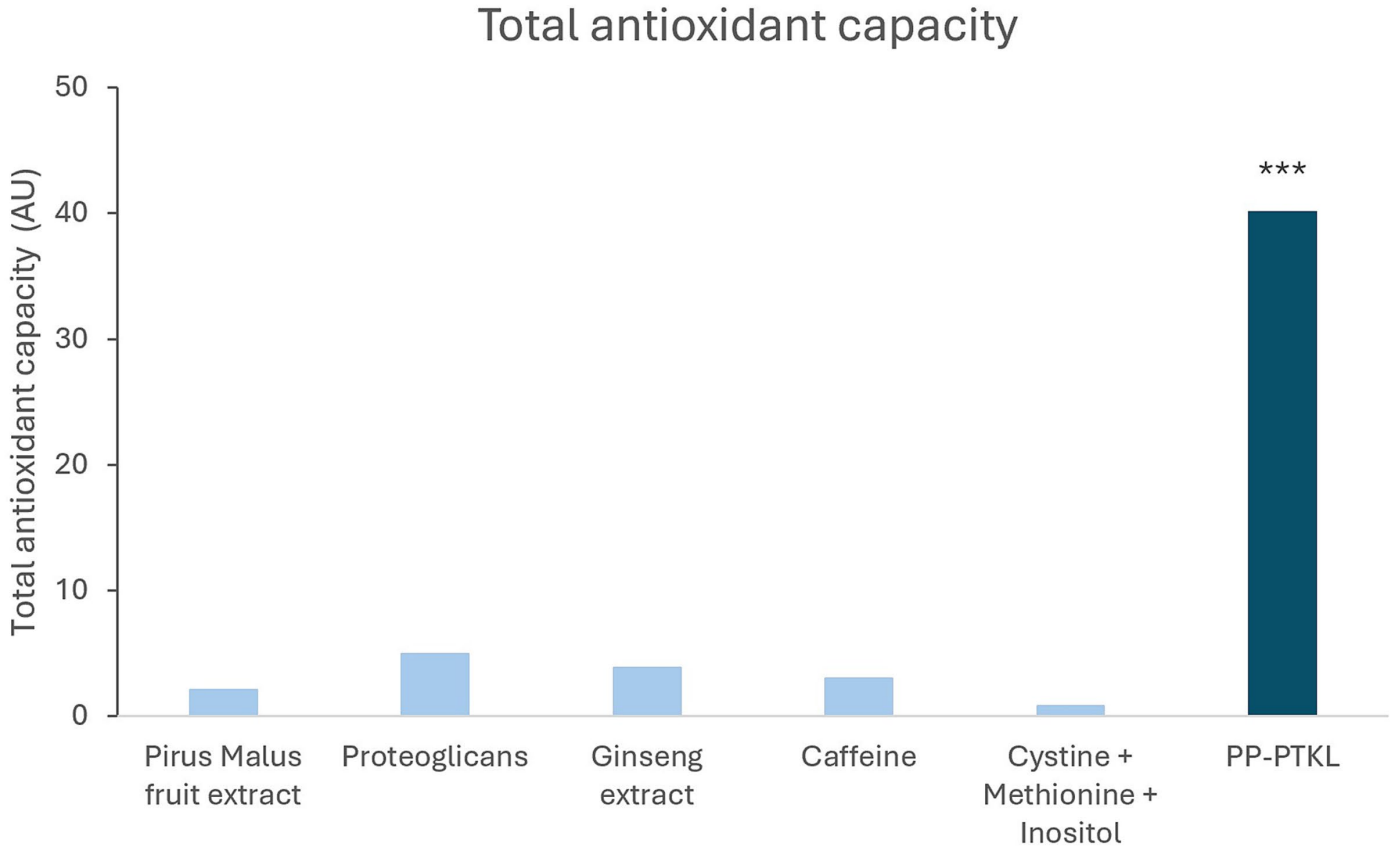
Total antioxidant capacity of PP-PTK and its active ingredients. Asterisks (*) over bars indicate statistical significance vs. Control.

When measuring total antioxidant capacity (TAC), every component of PP-PTKL exhibited intrinsic antioxidant capacity. The complete formulation demonstrated a synergistic effect, with total antioxidant capacity nearly four times greater than the sum of its individual component (Figure 2).

### Histamine, IL1-Ra and S100 inhibition

**Figure 3:**
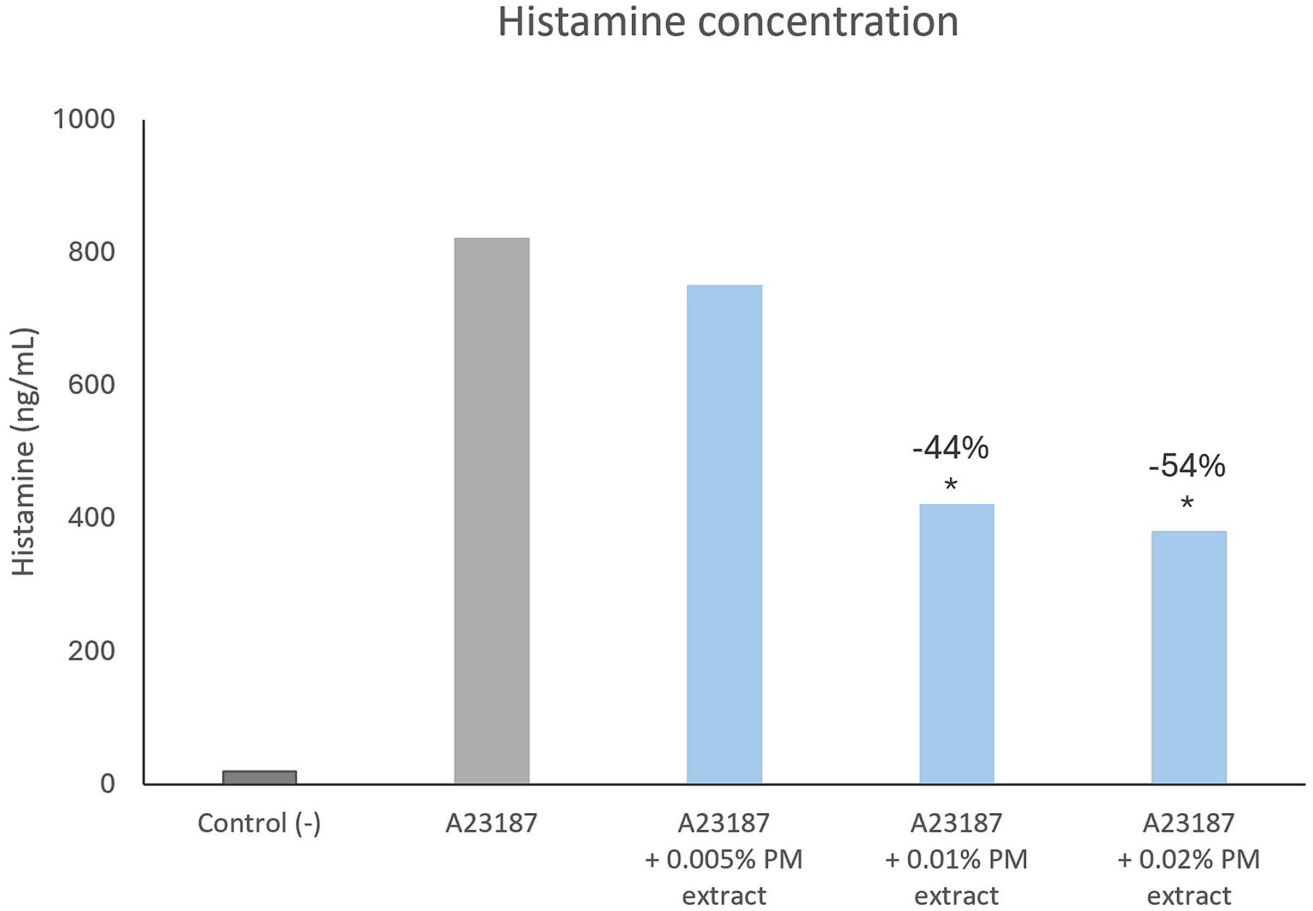
*In vitro* inhibition of histamine release by mast cells (*p* < 0.05). Asterisks (*) over bars indicate statistical significance vs. Control.

When treated with the *pyrus malus* extract, mast cells released significantly less histamine in a dose-dependent manner, as shown in Figure 3.

**Figure 4:**
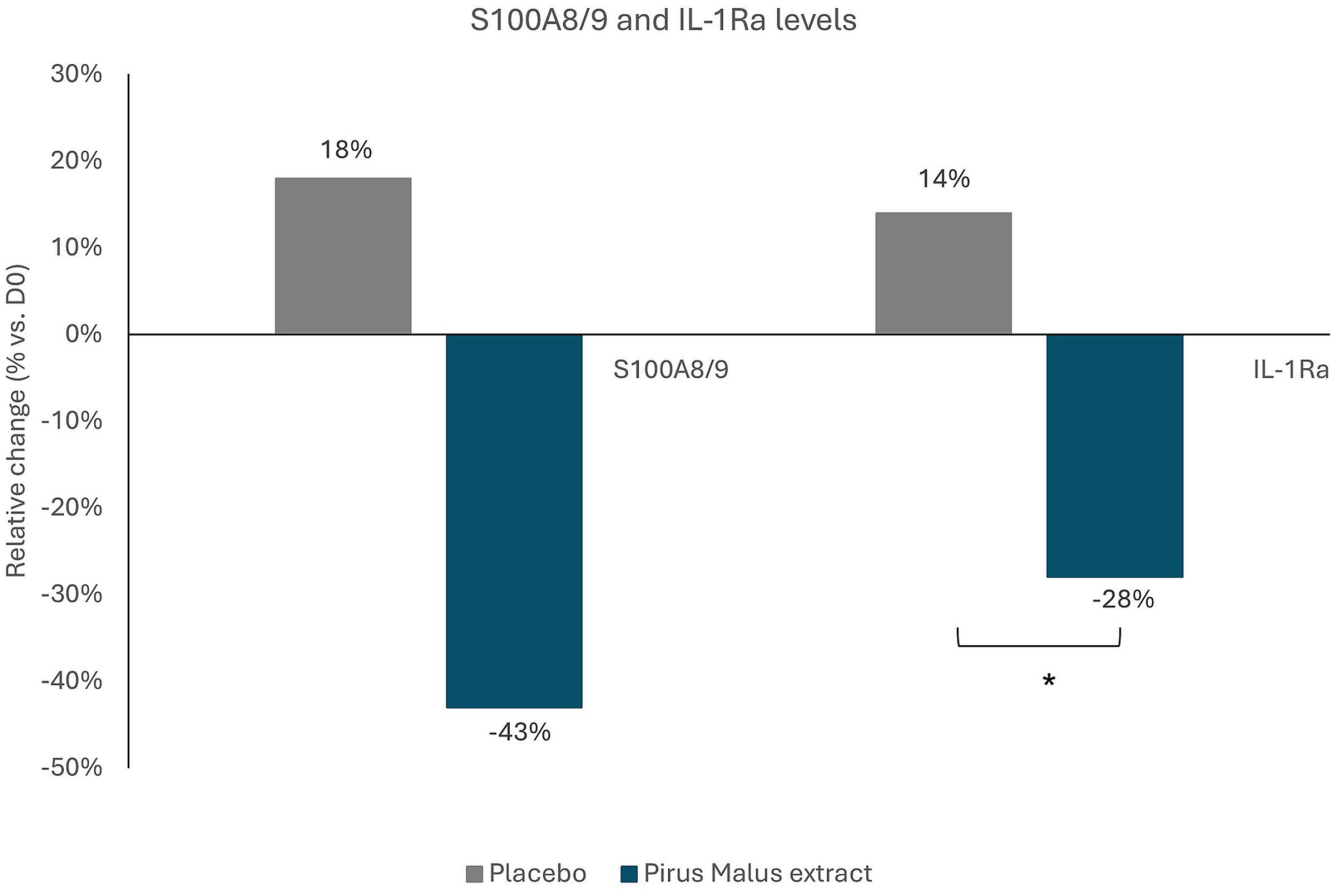
Variation of S100A8/9 and IL-1Ra levels after use of *Pyrus malus* extract (green) or placebo (grey) relatively to day 0. Measurements were done by ELISA from swab samples (*p* < 0.05). Asterisks (*) over bars indicate statistical significance vs. Control.

Both S100A8/9 and IL-1Ra levels were significantly reduced in the active ingredient group compared with the placebo (Figure 4).

## Discussion

Hair greying is increasingly recognized as a hallmark of hair aging, driven by cumulative oxidative stress, mitochondrial dysfunction, and progressive loss or dysfunction of melanocytes and melanocyte stem cells (7, 24). In contrast to androgenetic alopecia, senescent hair aging is molecularly characterized by impaired redox homeostasis and altered cellular stress responses highlighting the need for targeted anti-aging strategies (7, 25).

In this study, PP-PTKL demonstrated a dual biological activity relevant to hair greying: stimulation of melanocytic function and attenuation of oxidative and inflammatory stress. Inhibition of Wnt/β-catenin signaling markedly suppressed MITF expression, confirming the central role of this pathway in melanocyte activity (9–12). Treatment with PP-PTKL significantly restored MITF expression in a dose-dependent manner, reaching a 3.7-fold increase at the highest concentration tested. Given that reduced or absent MITF expression has been reported in grey hair follicles (16), these findings support MITF reactivation as a relevant mechanistic target for delaying pigment loss (12–18).

Oxidative stress is a key contributor to melanocyte dysfunction and hair greying progression (26, 27). Consistent with this, PP-PTKL exhibited strong total antioxidant capacity, exceeding the additive effect of its individual components. In parallel, the formulation significantly reduced inflammatory mediators associated with oxidative stress, including S100A8/9 and IL-1Ra (28). Overexpression of these proteins is known to amplify ROS production and inflammatory signaling via NADPH oxidase activation and downstream COX-2 and NOS induction, ultimately impairing keratinocyte proliferation and melanogenesis and promoting hair greying (29). Downregulation of these pathways supports PP-PTKL’s dual antioxidant and anti-inflammatory activity as a mechanism to preserve hair pigmentation, in line with previous evidence that ROS neutralization combined with melanocyte activation may delay hair greying (30).

Stress-related inflammatory pathways may further contribute to hair greying through histamine-mediated mechanisms. During acute physical or psychological stress, noradrenaline release from the sympathetic nervous system can induce rapid melanocyte depletion, leading to hair greying (31). Notably, this process has been shown to be partially reversible upon stress alleviation, coinciding with upregulation of antioxidant and energy metabolism pathways (32). Histamine release from mast cells, which may occur during neurogenic inflammation mediated by substance P, further amplifies inflammatory responses (33–35). In this context, *Pyrus malus* extract significantly reduced histamine release *in vitro*, suggesting an additional anti-inflammatory mechanism. Moreover, heparan-sulfate proteoglycans such as decorin and versican have been reported to inhibit mast cell degranulation and histamine release (36), reinforcing the biological relevance of their inclusion in the formulation.

Beyond pigmentation, proteoglycans play a central role in follicular homeostasis by promoting anagen induction, reducing catagen transition, and protecting follicular cells from oxidative stress and apoptosis (23, 36) Their levels have been shown to correlate with melanocyte pigmentation, with deficiency leading to pigment loss (37). These findings support a multifactorial mechanism by which PP-PTKL may contribute to delaying hair aging.

The main limitation of this work is its *in vitro* design, which warrants confirmation through further studies to validate these findings and assess their potential clinical relevance in hair aging therapies. Nonetheless, the combined antioxidant, anti-inflammatory, and melanocyte-stimulating effects of PP-PTKL provide a compelling rationale for further exploration as a preventive or therapeutic approach to hair aging and greying.

## Conclusion

Our *in vitro* results demonstrate that PP-PTKL exerts pro-melanocytic activity through MITF upregulation, together with strong total antioxidant capacity and suppression of inflammatory mediators including S100A8/9, histamine, and IL-1. These findings support a synergistic antioxidant and pro-melanogenic effect that promotes melanocyte survival and function, highlighting its potential as a preventive or therapeutic strategy for hair aging and greying. Future *in vivo* and clinical studies are needed to confirm these effects and define their therapeutic relevance.

## Data Availability

The raw data supporting the conclusions of this article will be made available by the authors, without undue reservation.
